# Antimicrobial resistance and interspecies gene transfer in *Campylobacter coli* and *Campylobacter jejuni* isolated from food animals, poultry processing, and retail meat in North Carolina, 2018–2019

**DOI:** 10.1371/journal.pone.0246571

**Published:** 2021-02-11

**Authors:** Dawn M. Hull, Erin Harrell, Arnoud H. M. van Vliet, Maria Correa, Siddhartha Thakur

**Affiliations:** 1 Department of Population Health and Pathobiology, College of Veterinary Medicine, North Carolina State University, Raleigh, North Carolina, United States of America; 2 School of Veterinary Medicine, University of Surrey, Guildford, Surrey, United Kingdom; Cornell University, UNITED STATES

## Abstract

The Center for Disease Control and Prevention identifies antimicrobial resistant (AMR) *Campylobacter* as a serious threat to U.S. public health due to high community burden, increased transmissibility, and limited treatability. The National Antimicrobial Resistance Monitoring System (NARMS) plays an important role in surveillance of AMR bacterial pathogens in humans, food animals and retail meats. This study investigated *C*. *coli* and *C*. *jejuni* from live food animals, poultry carcasses at production, and retail meat in North Carolina between January 2018-December 2019. Whole genome sequencing and bioinformatics were used for phenotypic and genotypic characterization to compare AMR profiles, virulence factors associated with Guillain-Barré Syndrome (GBS) (*neuABC* and *cst-II* or *cst-III*), and phylogenic linkage between 541 *Campylobacter* isolates (*C*. *coli* n = 343, *C*. *jejuni* n = 198). Overall, 90.4% (489/541) *Campylobacter* isolates tested positive for AMR genes, while 43% (233/541) carried resistance genes for three or more antibiotic classes and were classified molecularly multidrug resistant. AMR gene frequencies were highest against tetracyclines (64.3%), beta-lactams (63.6%), aminoglycosides (38.6%), macrolides (34.8%), quinolones (24.4%), lincosamides (13.5%), and streptothricins (5%). A total of 57.6% (114/198) *C*. *jejuni* carried GBS virulence factors, while three *C*. *coli* carried the *C*. *jejuni*-like lipooligosaccharide locus, *neu*ABC *and cst-II*. Further evidence of *C*. *coli* and *C*. *jejuni* interspecies genomic exchange was observed in identical multilocus sequence typing, shared sequence type (ST) 7818 clonal complex 828, and identical species-indicator genes *mapA*, *ceuE*, and *hipO*. There was a significant increase in novel STs from 2018 to 2019 (2 in 2018 and 21 in 2019, p<0.002), illustrating variable *Campylobacter* genomes within food animal production. Introgression between *C*. *coli* and *C*. *jejuni* may aid pathogen adaption, lead to higher AMR and increase *Campylobacter* persistence in food processing. Future studies should further characterize interspecies gene transfer and evolutionary trends in food animal production to track evolving risks to public health.

## Introduction

*Campylobacter* is a gram-negative commensal bacterium in the gastrointestinal tract of multiple wild and domesticated animal species [1]. According to the Center for Disease Control and Prevention (CDC), *Campylobacter* is estimated to cause 1.3 million cases of human illness in the United States annually [2]. In 2019, *Campylobacter* was the leading cause of U.S. foodborne illness, with an overall incidence of 19.5 per 100,000 population [3]. The incidence of human campylobacteriosis cases in the U.S. progressed in 2019 despite targeted efforts to reduce the pathogen in the food supply. The Foodborne Diseases Active Surveillance Network (FoodNet) of the CDC’s Emerging Infections Program reported a 13% increase (confidence interval (CI) 5–21) in incidence compared to cases in 2016–2018 [3]. *Campylobacter* contaminated food and water are common sources for human illness worldwide. Specifically, the consumption of poultry products are a known risk factor for human *Campylobacter* infections in the U.S and in other parts of the world. [1, 4–6]. Food animals (poultry, cattle and swine) are common asymptomatic reservoir hosts for pathogenic *Campylobacter* strains. *C*. *coli* and *C*. *jejuni* coexist in large numbers in animal gastrointestinal tracts and within the environment by manure contamination [5, 7–9]. *Campylobacter* are known to have a hypervariable genome with evidence of interspecies genomic exchange [9]. Previous studies have shown some *C*. *coli* lineages have been progressively accumulating *C*. *jejuni* DNA [10]. Interspecies horizontal gene transfer (HGT) between *C*. *coli* and *C*. *jejuni* is presumed to aid pathogen adaption and persistence in austere conditions [9, 11]. A recent study determined *C*. *coli* and *C*. *jejuni* HGT can create hybrid strains which may evade traditional diagnostic methods like qPCR due to the alteration of species-specific target genes [11]. As far as we know, evidence of *Campylobacter spp*. hybridization has not been previously reported in the NC food animal processing environment.

The U.S. NARMS program began conducting surveillance of foodborne pathogens in 1996 among human illness, retail meat, and food animals. NARMS samples are routinely collected by the CDC, Food and Drug Administration (FDA), and U.S. Department of Agriculture (USDA) respectively with the assistance of partner agencies to provide trends in emerging antimicrobial resistance [12, 13]. Antibiotic resistance among bacterial pathogens is one of the world’s growing public health threats [14]. Decades of antibiotic use in humans, animals, and agriculture have created selection pressures driving the emergence of AMR in many bacterial pathogens and an increase in clinical infections that do not respond to routine medical intervention [5, 15]. Despite recent policy changes and restricted antibiotic use in some countries, drug applications in human and animal populations, environmental contamination, and increased globalization continue to steer selection pressures and foster opportunities for horizontal gene transfer among AMR bacteria [14, 16]. The World Health Organization (WHO) highlights fluoroquinolone resistant *C*. *coli* and *C*. *jejuni* as high priority pathogens in efforts to ignite and focus global research and development of new antibiotic strategies [15]. *Campylobacter* antimicrobial resistance is increasing in high-income, low- and middle-income countries around the world [15].

Campylobacteriosis infections can also trigger debilitating chronic health conditions such as reactive arthritis, post-infectious irritable bowel syndrome, carditis, endocarditis, cholecystitis, pancreatitis, meningitis, septicemia and an autoimmune-mediated demyelinating neuropathy disorder called Guillain-Barré Syndrome (GBS), which often presents with ascending weakness, sensory loss, autonomic dysfunction, paralysis, bulbar palsy and respiratory insufficiency with a 5% mortality rate [17–20]. Human campylobacteriosis is often treated with macrolide antimicrobials, such as erythromycin, while ciprofloxacin, a fluoroquinolone antimicrobial, is often used for generalized cases of traveler’s diarrhea or gastroenteritis [20–23]. Drug resistant *Campylobacter* can impede effective treatment of routine infections and result in critical outcomes [24].

*Campylobacter* can also survive many sanitary interventions in food production. U.S. poultry processing incorporates scalding, washing, and chilling which are effective at reducing bacterial counts but do not eliminate all pathogenic bacteria from the finished meat products [7]. The animal carcass liquid film and skin crevasses provide a suitable microenvironment for *Campylobacter* survival during production [1, 5]. Previous studies found *Campylobacter* contaminates approximately 50% of retail chicken meat in the U.S., and significantly less was found in other retail meat products [25]. This study compares *C*. *coli* and *C*. *jejuni* from NARMS isolates in three stages of meat production including, live food animals, poultry carcasses at production, and retail meat sold in grocery stores in North Carolina from 2018 and 2019. Processed meat and retail meat samples in this study included chicken and turkey only; processed pork and beef were not sampled. The main objectives met in this study were to characterize AMR phenotypes and genotypes, to evaluate virulence factor distribution associated with GBS, to determine the phylogenic linkage, and to assess interspecies genome exchange between the *Campylobacter* isolates.

## Results

### Dataset

Evaluation of the 541 *Campylobacter* isolates in this dataset revealed an uneven *C*. *coli* and *C*. *jejuni* distribution across different sources. *C*. *coli* comprised all isolates from live turkey, 95% comminuted turkey (21/22) and 94% swine (66/70), while *C*. *jejuni* was the main species identified in 76% cattle (17/22) isolates. The prominent *Campylobacter spp*. isolated from chicken in early stages of production differed from those isolated from finished retail meat products. Most isolates from live chicken and chicken carcasses were C. coli at 54% (38/71) and 60% (162/270) respectively, while C. jejuni was the prominent species in retail chicken at 69% (36/52). This difference was significant as chicken retail meat *Campylobacter* isolates were three times more likely to be *C*. *jejuni* compared to isolates from live chickens and chicken carcasses sampled in processing facilities [OR: 3.0 (95% CI 1.6–5.7) P = 0.0004].

### AMR phenotype and genotype

#### Phenotypic AMR analysis antibiotic susceptibility testing (AST)

**Fig 1:**
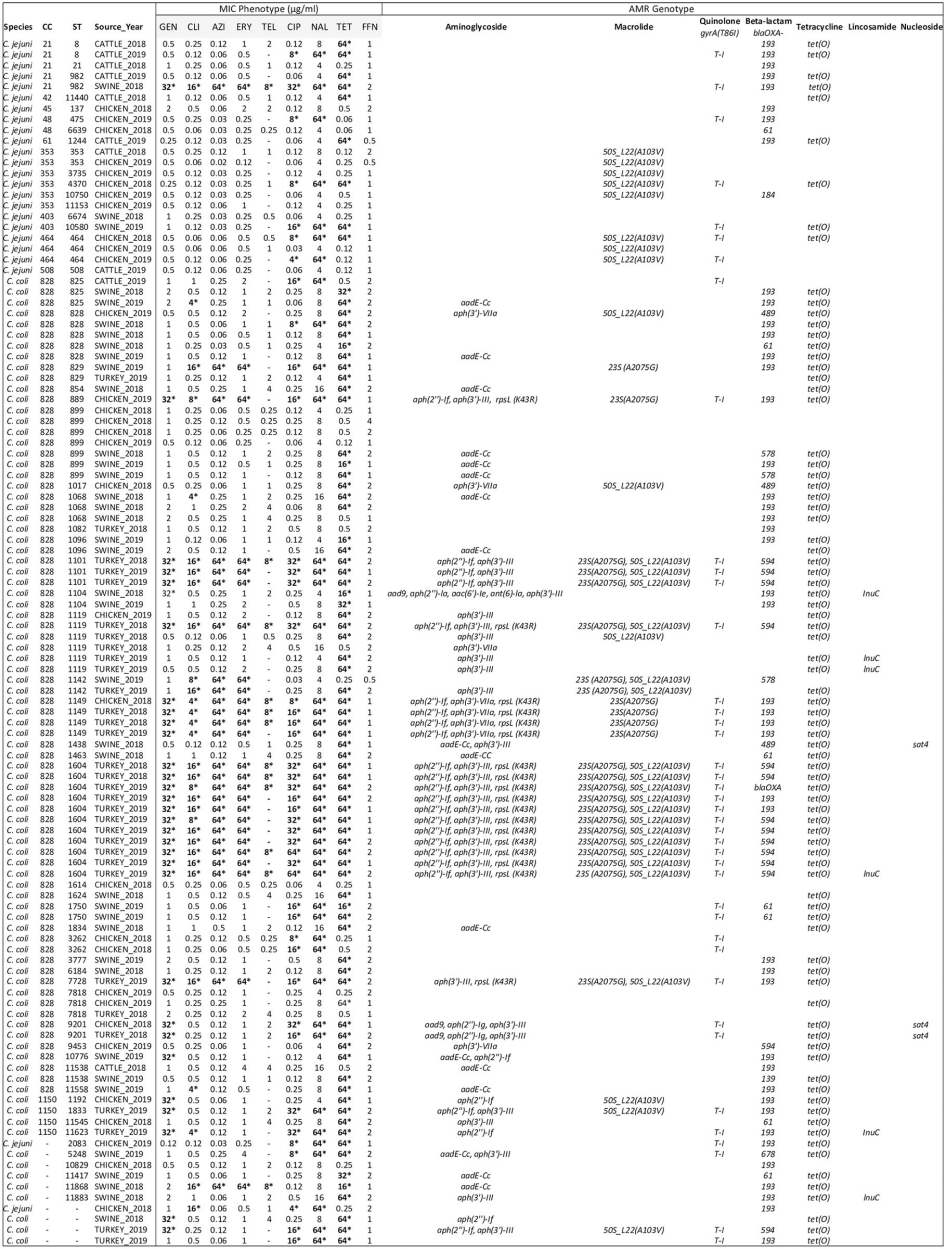
*Campylobacter* antibiotic susceptibility test results in live food animals aligned with AMR genotype, MLST, and source. Bold (*) = resistant. NARMS USDA-FSIS *C*. *coli and C*. *jejuni* minimum inhibitory concentration (MIC) results in μg/ml concentration for live food animals in North Carolina compared with MLST and AMR genotype. MIC was determined for the following antibiotics: Gentamicin (GEN), Clindamycin (CLI), Azithromycin (AZI), Erythromycin (ERY), Telithromycin (TEL), Ciprofloxacin (CIP), Nalidixic Acid (NAL), Tetracycline (TET), and Florfenicol (FFN).

A representative group of *Campylobacter* isolates from live animal samples were selected for antimicrobial susceptibility testing (AST) by the USDA NARMS laboratory. Antimicrobial minimum inhibitory concentrations (MIC) were determined for a total of 111 *Campylobacter* isolates from live chicken (n = 31), turkey (n = 32), swine (n = 38), and cattle (n = 10) for ten medically relevant antibiotics from seven drug classes (Fig 1). The AST revealed twenty-two (19.8%) pan-susceptible isolates, including 13.8% (12/87) *C*. *coli* and 42.7% (10/24) *C*. *jejuni*. The highest frequency of resistance observed was against tetracycline (74%) which corresponded to 99% (82/83) *tet(O)* AMR gene expression. All isolates containing *tet(O)* had high resistance to tetracycline (≥ 64 μg/ml). In only one tetracycline resistant isolate, the tet(O) gene was not identified. Quinolone resistance, for both nalidixic acid and ciprofloxacin, was expressed in 39.6% (44/111) isolates. The *gyrA* mutation was identified in 93% (41/44) of the quinolone resistant isolates. Three isolates resistant to nalidixic acid and ciprofloxacin did not have a quinolone resistance genotype identified.

All *Campylobacter* isolates expressing macrolide resistance were resistant to both azithromycin and erythromycin. Macrolide resistance was expressed in 28.7% (25/87) *C*. *coli* and 4.2% (1/24) *C*. *jejuni*. The *23S (A2075G)* mutation was detected in 96% (24/25) macrolide resistant *C*. *coli*. We were unable to identify a specific corresponding predictive genotype in the remaining two macrolide resistant isolates (1 *C*. *coli* and 1 *C*. *jejuni* collected from swine in 2018). The sole presence of *50S_L22 (A103V)* was not associated with azithromycin and erythromycin resistance. All 14 *Campylobacter* isolates carrying the *50S_L22 (A103V)* mutation were susceptible to both macrolide antibiotics tested. Clindamycin resistance was observed in 33.3% (29/87) *C*. *coli* and 8.3% (2/24) *C*. *jejuni*, with 77% (24/31) of the clindamycin resistant isolates containing the *23S (A2075G)* mutation, 2 carrying the *lnuC* gene and 19% (6/31) lacking a specific lincosamide AMR profile. Four out of the six (67%) isolates carrying *lnuC* were found to be susceptible to clindamycin. 34.5% (30/87) *C*. *coli* and 4.2% (1/24) *C*. *jejuni* expressed resistance to gentamicin. A single isolate, out of the 31 gentamicin resistant isolates, did not have a corresponding aminoglycoside resistance gene identified (Fig 1).

**Fig 2:**
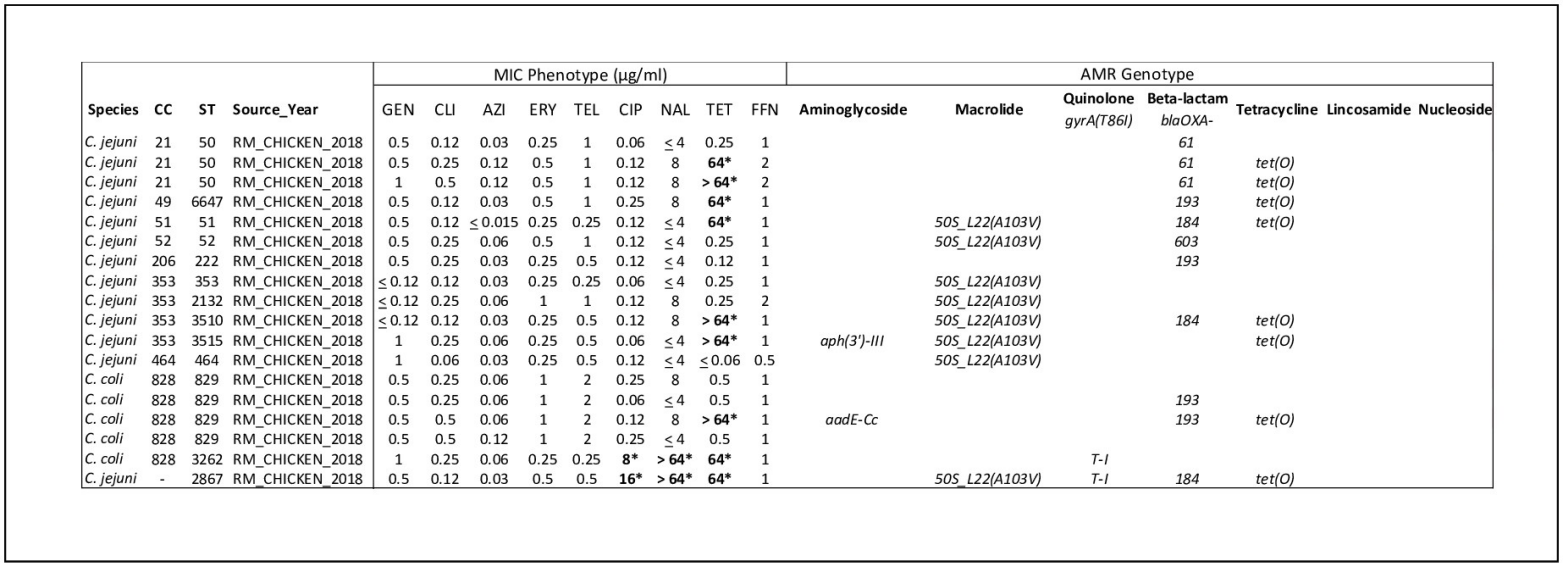
*Campylobacter* antibiotic susceptibility test results in retail meat aligned with AMR genotype, MLST, and source. Bold (*) = resistant. NARMS FDA *C*. *coli and C*. *jejuni* minimum inhibitory concentration (MIC) results for retail meat in North Carolina compared to isolate MLST and AMR genotype. MIC was determined for the following antibiotics: Gentamicin (GEN), Clindamycin (CLI), Azithromycin (AZI), Erythromycin (ERY), Telithromycin (TEL), Ciprofloxacin (CIP), Nalidixic Acid (NAL), Tetracycline (TET), and Florfenicol (FFN).

AST was also conducted on all of the NC 2018 retail meat isolates at the FDA NARMS laboratory and resulted in phenotypic AMR MIC for 18 retail meat isolates (Fig 2). Half of the isolates were pan-susceptible, while the other half (9/18) expressed resistance to tetracycline. All but one tetracycline resistant isolates contained *tet(O)*. 11% (2/18) also expressed quinolone resistance to both nalidixic acid and ciprofloxacin. The two quinolone resistant isolates contained the *gyrA(T86I)* mutation (Fig 2).

#### Whole Genome Sequencing (WGS) based AMR analysis

The genomic assemblies for all 541 *Campylobacter* isolates screened for AMR genes revealed 90.4% (489/541) *C*. *coli and C*. *jejuni* contained at least one AMR gene while 43.1% (233/541) contained resistance genes to three or more antibiotic drug classes and were classified as molecularly MDR. The prevalence of molecular MDR isolates varied significantly by *Campylobacter* species. *C*. *coli* were twice as likely to carry resistance genes to three or more antibiotic classes compared to *C*. *jejuni* [OR 1.9 (CI 1.3–2.7), p = 0.0006]. Related to this finding, *Campylobacter* isolated from turkey were 13 times more likely to carry an MDR genotypic profile compared to isolates from chicken [OR 13.2 (CI 5.8–29.9), p < .0001]. Within the turkey dataset, the prevalence of MDR isolates was lower in live animals (83%) compared to meat processing (95%). Although the MDR prevalence from comminuted turkey at production tended to be higher than isolates from live turkey samples, the difference between the two sources was not significant (P >0.05) (S1 Fig). Differences from 2018 to 2019 in AMR and MDR for both *C*. *coli* and *C*. *jejuni*, overall and within the sampling categories, were also not significant (P>0.05).

The MDR genomic profiles present in this dataset are summarized in S1 Table. One genomic AMR consideration not displayed in the table, which could alter the predicted phenotype, is the *Campylobacter* multidrug efflux complex, *cmeABC*, *cmeR*, and associated mutations within this complex which may promote a drug resistant phenotype. The majority of *C*. *coli and C*. *jejuni* isolates in this dataset contained *cmeABC* and *cmeR* genes with >90% coverage and a variable percent identity (range 75.9–100%).

Overall, *Campylobacter* molecular analysis predicted the following AMR prevalence by drug class: 64.3% tetracyclines, 63.6% beta-lactams, 38.6% aminoglycosides, 34.8% macrolides, 24.4% quinolones, 13.5% lincosamides, and 5% streptothricins resistance. Resistance predictions for tetracyclines and streptothricins were predicted 100% by the presence of *tet(O)* and *sat4* genes, respectively. Tetracycline resistance genes were found in both *C*. *coli and C*. *jejuni* from every source, with swine carrying the highest *tet(O)* prevalence at 94% (66/70). *Sat4* appeared to be carried more often by *C*. *coli* but this observed difference between *C*. *coli* and *C*. *jejuni* was not significant (p = 0.06). Beta-lactam genes were carried in 63.6% of *Campylobacter* isolates in this dataset. The diversity of beta-lactam genes was widespread with the identification of 12 different beta-lactam AMR gene variations spanning both *Campylobacter* species and all sample categories.

**Fig 3:**
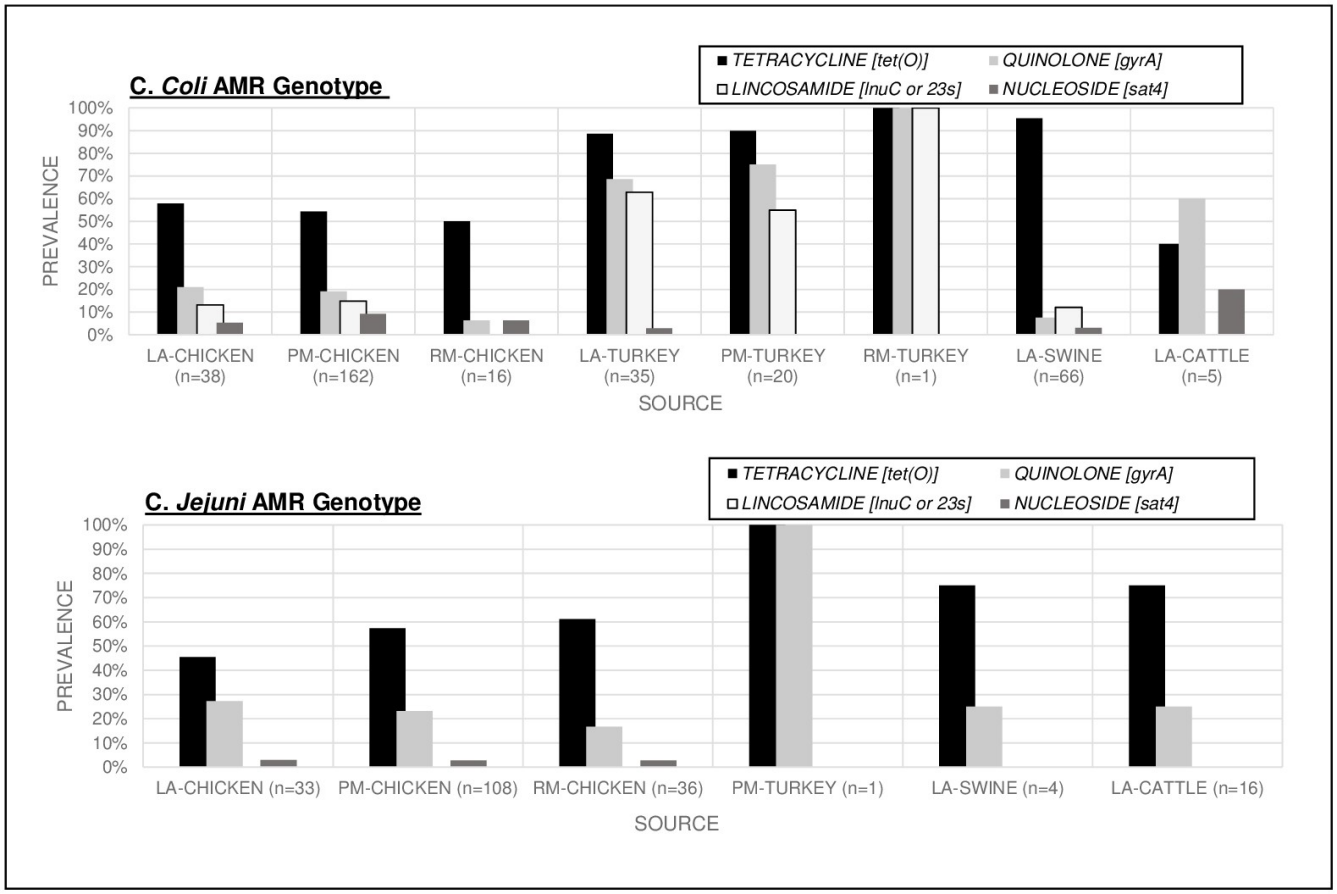
*C*. *coli* and *C*. *jejuni* AMR gene prevalence by source conferring resistance to tetracyclines, quinolones, lincosamides, and nucleosides. Chart coding: Live Animal (LA), Processed Meat (PM), Retail Meat (RM).

Quinolone resistance was conferred by the point mutation *gyrA(T86I)* and one *C*. *coli* isolate from comminuted chicken contained the *gyrA(D90N)* mutation. *gyrA(T86I)* was detected in both *C*. *coli and C*. *jejuni* from all sources (Fig 3). The highest prevalence of quinolone resistance was 71.4% in *C*. *coli* in turkey isolates (procured from both live and comminuted samples). *C*. *jejuni* isolates in chicken were 22.6% resistant, which was slightly higher than the *C*. *coli* isolated from chicken. The prevalence of this point mutation was not significantly different between *C*. *coli and C*. *jejuni* (25.7% and 23.2% respectively). However, it was found that turkey *C*. *coli* isolates were 10 times more likely to carry the *gyrA(T86I)* point mutation compared to *C*. *coli* isolated from chicken [OR10.3, CI 5.5–19.2, (p<0.001)].

**Fig 4:**
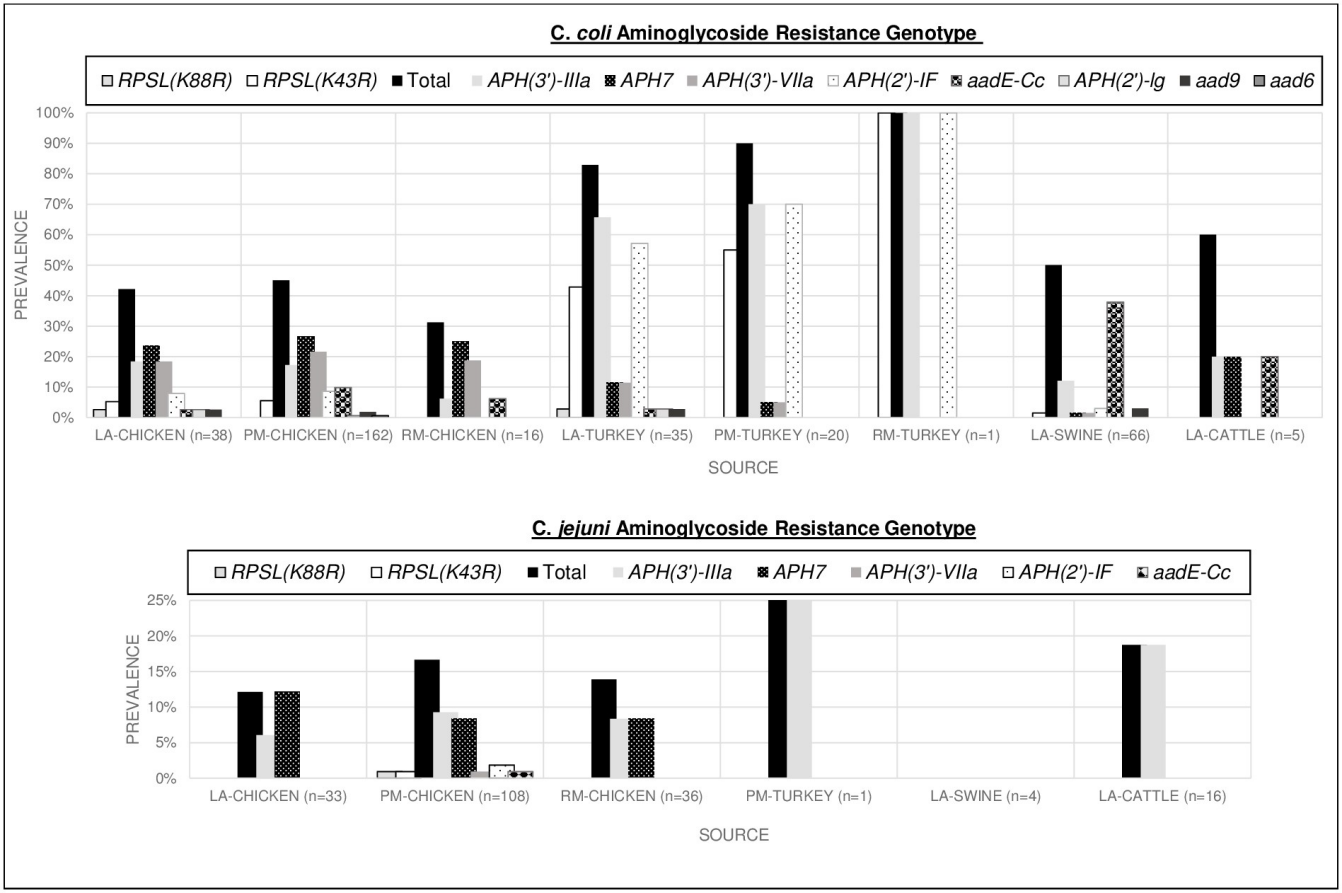
*C*. *coli* and *C*. *jejuni* AMR gene prevalence by source conferring aminoglycoside resistance. AMR genes inferring resistance to the following antimicrobials: *aad6*: streptomycin; *aad9*: aminoglycosides (specific antibiotics are not listed in the database for this gene); *aadE-Cc*: streptomycin; *aph(2’)-If*: amikacin, gentamicin, kanamycin, tobramycin; *aph(2’)-lg*: amikacin, gentamicin, kanamycin, tobramycin; *aph(3’)-IIIa*: amikacin, kanamycin; *aph(3’)-VIIa*: amikacin, kanamycin; *aph7*: hygromycin. Chart coding: Live Animal (LA), Processed Meat (PM), Retail Meat (RM).

Aminoglycoside resistance gene diversity was greater among *C*. *coli* with 8 different aminoglycoside resistance genes and two point mutations detected. *C*. *coli* isolates were 6 times more likely to contain an aminoglycoside resistance genotype compared to *C*. *jejuni* [OR 5.8, CI 3.7–9.0 (p<0.001)]. *C*. *coli* were more likely to contain aminoglycoside genes *aad9*, *aadE-Cc*, *aph(2’)-If*, *aph(3’)-IIIa*, *aph(3’)-VIIa* (conferring resistance to streptomycin, amikacin, gentamicin, kanamycin, tobramycin) and 25 times more likely to have the *rpsl(K43R)* mutation [OR 25.2, CI 3.4–185.45, (p<0.001)] predicted resistance to streptomycin. The *C*. *coli* aminoglycoside resistance genes were found across different sources and all stages of production. *C*. *jejuni* isolates primarily only carried 2 aminoglycoside resistance genes, *aph7* and *aph(3’)-IIIa*, which confer resistance to hygromycin, amikacin and kanamycin. The greatest *C*. *jejuni* aminoglycoside resistance gene diversity was found in chicken carcasses at production. Details of aminoglycoside AMR gene prevalence by source are provided in Fig 4.

**Fig 5:**
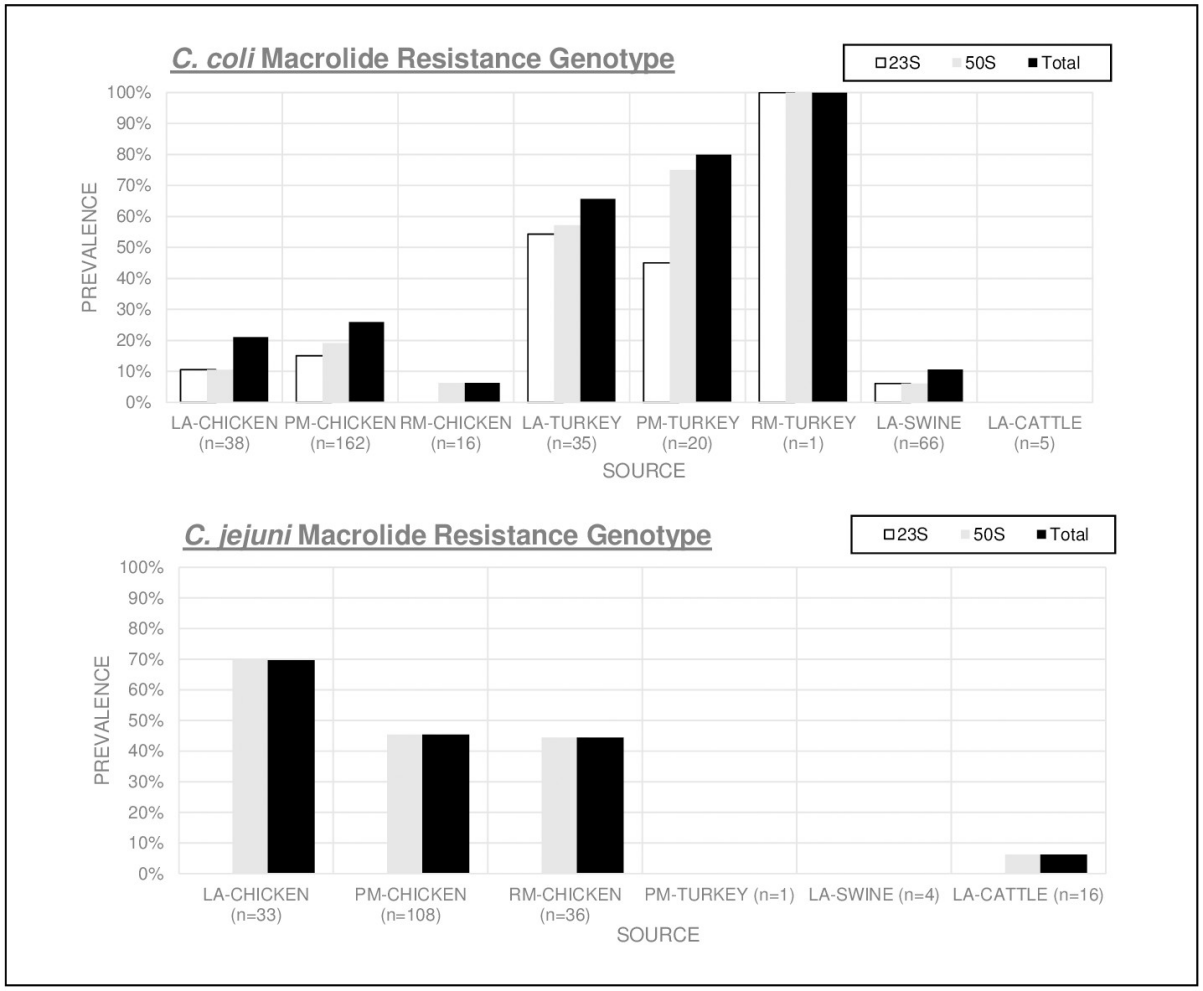
*C*. *coli* and *C*. *jejuni* AMR gene prevalence by source conferring macrolide resistance. Chart coding: Live Animal (LA), Processed Meat (PM), Retail Meat (RM).

The mutations inferring macrolide resistance identified were *23S (A2075G)*, *50S L22 (A103V)*, *and 50S L22 (G86E)* (Fig 5). The *23S (A2075G)* mutation was discovered only in *C*. *coli* isolates, while *C*. *jejuni* were three times more likely to have the *50S L22* mutation [OR 2.9, CI 2.0–4.2 (p<0.0001)]. *C*. *coli* isolated from turkey had the highest mutation prevalence of 52% (29/56) for *23S (A2075G)* and 64% (36/56) for *50S L22 (A103V)* with 45% containing both mutations. The overall *Campylobacter* macrolide resistance genotype prevalence in turkey *C*. *coli* was 71% (40/56). The results show turkey *C*. *coli* isolates were 9 times more likely than *C*. *coli* from chicken and 59 times more likely than *C*. *coli* from live swine, to contain either mutation which infers potential resistance to macrolide antibiotics [OR 9.2, CI 4.7–17.9 (p<0.0001) and OR 58.9 CI 22.7–152.8 (p<0.001) respectively].

### GBS virulence factors

In this dataset (541 *Campylobacter* isolates), primarily *C*. *jejuni* from chicken and cattle contained the genetic machinery for LOS glycan mimicry and qualified as potential etiological agents for GBS. The associated virulence factors *neuABC* and *cst-II* or *cst-III* were carried in 57.6% (114/198) of all *C*. *jejuni*. Of these, 30.8% (61/198) contained *cst-II* and 26.8% (53/198) carried *cst-III*. *Cst-III* LOS virulence factors appeared to be more reserved to specific *C*. *jejuni* multilocus sequence types (ST) and clonal complexes (CC). 100% ST 8, ST 50, ST 536, and ST 10738 (represented by four or more isolates) contained *neuABC* and *cst-III* virulence factor prevalence and were components of CC 21 and CC 353. *Cst-II* had a wider distribution, with presence in over twice as many multilocus ST and CC as *cst-III* (S2 and S3 Tables). *Cst-II* was also found within 3 *C*. *coli* with > 90% coverage and identity. The majority of *Campylobacter* isolates with both GBS virulence factors were procured from processed chicken carcasses, live chicken and retail meat with 67% (72/108), 52% (17/33), and 39% (14/36) prevalence in the groups respectively, followed by 53% (9/17) in cattle.

### MLST phylogeny

**Fig 6:**
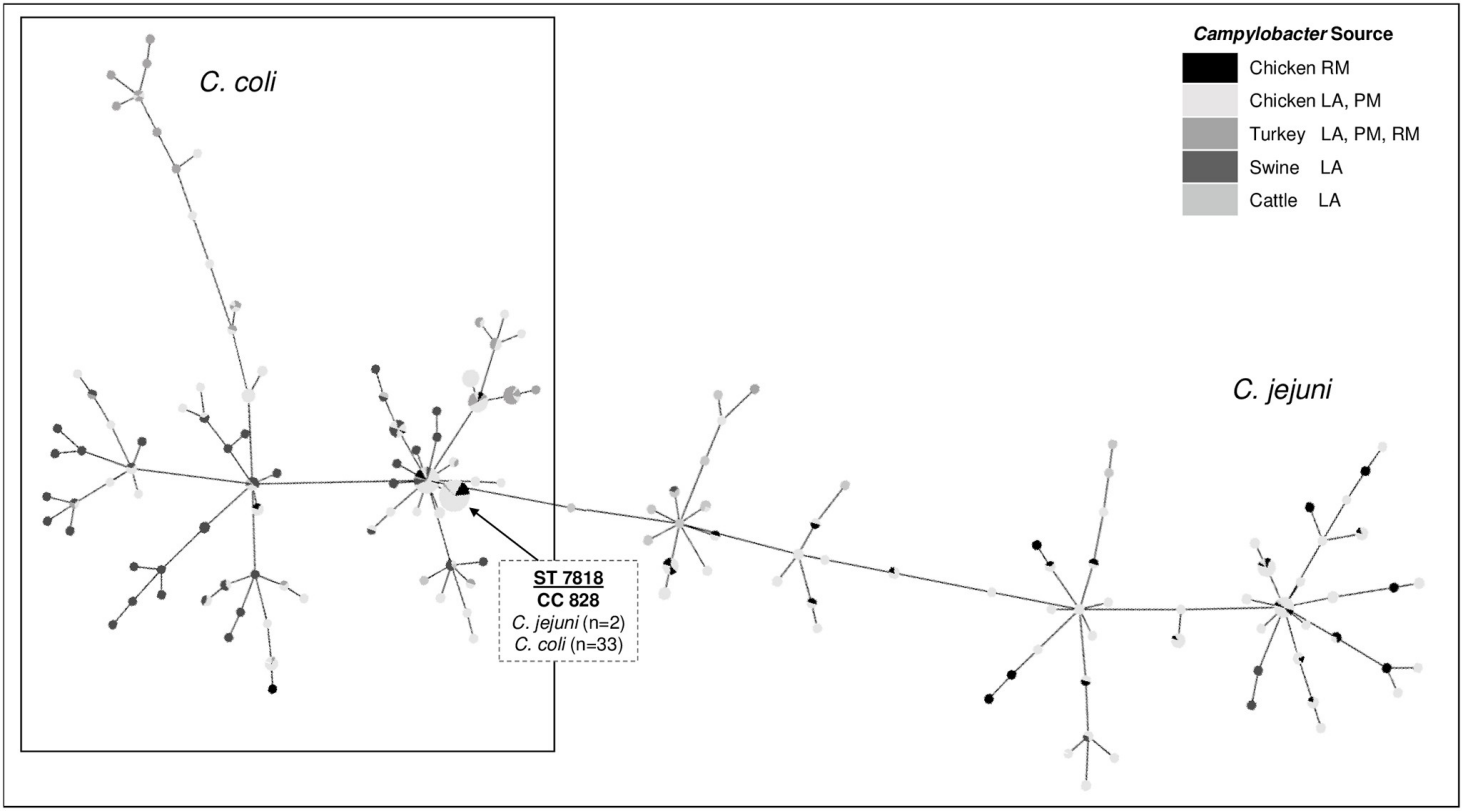
*C*. *coli* and *C*. *jejuni* MLST minimum spanning tree. Expansion generated by PHYLOVIZ software using an eBLAST algorithm. Link distances correlate to locus variations and represent the most likely relationship between isolates. Data points are colored by source according to the key. Isolates with unknown alleles were not included in the diagram.

This dataset of 343 *C*. *coli* and 198 *C*. *jejuni* isolates matched 144 ST within the MLST database (78 ST *C*. *coli* and 66 ST *C*. *jejuni)*. The MLST phylogeny diagram portrays the ancestral linkage between isolate ST based on the variation among the seven housekeeping alleles and provides an overview of ST distribution by source (Fig 6). A ST could not be generated for 4.3% (18 *C*. *coli* and 5 *C*. *jejuni)* due to an untypeable loci combination which represent novel ST (Table 1). For several of these isolates, one genetic variation of the seven housekeeping alleles did not match the reference pubMLST database (Table 1). There was a significant increase in untypeable sequences from 2018 to 2019, with only two collected in 2018 and 21 collected in 2019 (p<0.002). Most untypeable *Campylobacter* came from chicken, with one unknown ST *C*. *jejuni* present in 2018 chicken isolates to 17 undetermined ST (13 *C*. *coli* and 4 *C*. *jejuni*) among the 2019 chicken isolates (p = 0.002). The phylogeny analysis shows 78 *C*. *coli* ST were placed under two clonal complexes (CC) while the 66 *C*. *jejuni* ST were linked to 21 clonal complexes. A total of 88% (302/343) *C*. *coli* isolates fell within CC 828 while 4% (14/343) were linked to CC 1105. *C*. *jejuni* isolates were distributed widely across different CC with the majority belonging to CC 353 (39%, 78/198) and CC 21 (17.7%, 35/198). No significant differences in identified ST or CC prevalence were found in comparing isolates from 2018 to 2019 (p> 0.05). The 53 retail meat *Campylobacter* isolates (18 *C*. *coli* and 35 *C*. *jejuni)* represented strains viable and culturable despite adverse processing conditions and included 30 different STs. 100% retail meat *C*. *coli* ST belonged to CC 828 while *C*. *jejuni* spanned 11 different CC with most belonging to CC 353 (37%, 13/35) and CC 21 (17%, 6/35). Retail meat ST were clonal to 77% (23/30) *Campylobacter* isolated in earlier stages of poultry production.

**Table 1:** Novel ST MLST loci and allele matches for *C*. *Jejuni* and *C*. *coli* species specific genetic indicators.

		*Campylobacter* MLST Loci							CAMP0952	CAMP0908	CAMP1271
		*aspA*	*glnA*	*gltA*	*glyA*	*pgm*	*tkt*	*uncA*	*mapA*	*hipO*	*ceuE*
Species	Source_Year	Length: 477	Length: 477	Length: 402	Length: 507	Length: 498	Length: 459	Length: 489	Length: 645	Length: 1152	Length: 993
*C*. *coli*	LA_SWINE_2018	32	38	30	-	104	43	17	**-**	**-**	**163**
*C*. *jejuni*	LA_CHICKEN_2018	4	7	10	-	42	7	1	**1**	**2**	**5**
*C*. *jejuni*	PM_CHICKEN_2019	2	-	5	2	59	1	5	**24**	**16**	**1**
*C*. *jejuni*	PM_CHICKEN_2019	2	-	5	2	59	1	5	**24**	**16**	**1**
*C*. *jejuni*	PM_CHICKEN_2019	7	2	5	-	-	3	6	**21**	**22**	**32**
*C*. *jejuni*	LA_CHICKEN_2019	24	25	2	2	-	59	6	**29**	**45**	**109**
*C*. *coli*	LA_CHICKEN_2019	33	39	30	-	113	56	17	**3**	**-**	**3**
*C*. *coli*	LA_CHICKEN_2019	33	39	30	-	113	56	17	**3**	**-**	**3**
*C*. *coli*	LA_CHICKEN_2019	33	39	66	-	113	43	17	**3**	**-**	**3**
*C*. *coli*	PM_CHICKEN_2019	33	39	66	-	113	43	17	**3**	**-**	**3**
*C*. *coli*	PM_CHICKEN_2019	33	39	66	-	113	43	17	**3**	**-**	**3**
*C*. *coli*	PM_CHICKEN_2019	33	39	66	-	113	43	17	**3**	**-**	**3**
*C*. *coli*	PM_CHICKEN_2019	33	39	66	-	113	43	17	**3**	**-**	**3**
*C*. *coli*	PM_CHICKEN_2019	33	39	66	-	113	43	17	**3**	**-**	**3**
*C*. *coli*	PM_CHICKEN_2019	33	39	66	-	113	43	17	**3**	**-**	**3**
*C*. *coli*	PM_CHICKEN_2019	33	39	66	-	113	43	17	**3**	**-**	**3**
*C*. *coli*	PM_CHICKEN_2019	33	39	66	-	113	43	17	**3**	**-**	**3**
*C*. *coli*	PM_CHICKEN_2019	33	39	66	-	113	43	17	**3**	**-**	**3**
*C*. *coli*	RM_CHICKEN_2019	33	39	66	-	113	43	17	**3**	**-**	**3**
*C*. *coli*	LA_TURKEY_2019	103	110	103	140	188	172	79	**293**	**-**	**3**
*C*. *coli*	LA_TURKEY_2019	103	110	103	172	188	172	79	**293**	**-**	**999**
*C*. *coli*	LA_SWINE_2019	33	38	30	82	-	85	68	**3**	**-**	**16**
*C*. *coli*	LA_SWINE_2019	33	39	30	161	104	47	17	**3**	**-**	**3**

(-) indicates no genomic match within the database. The table details the alleles for the seven MLST housekeeping genes for each untypeable sequence. Allele numbers are also provided for matches to previously known species-specific genomic identifiers *mapA*, *hipO*, and *ceuE*.

The MLST diagram shows clonality between *C*. *coli* and *C*. *jejuni*, with a ST species crossover. Two *C*. *jejuni* collected in 2019 from chicken retail meat, contained the same MLST loci as 33 *C*. *coli* isolates (ST 7818, CC 828). In 2018, ST 7818 was only identified in live poultry and carcass samples at processing in 13 *C*. *coli*, while in 2019 ST 7818 was isolated from chicken at all three stages of poultry production. Species ST crossover was not observed in samples collected in 2018 nor for any other ST in this dataset. ST 7818 isolates also contained the same indicator genes *mapA* and *ceuE*, often used for PCR identification of *C*. *jejuni* and *C*. *coli*, respectively. The ST 7818 *C*. *jejuni* also did not carry the *hipO* gene, which is also targeted to differentiate the two species (Table 2).

**Table 2:** ST 7818 *Campylobacter* housekeeping allele MLST ST species crossover and allele matches for *C*. *Jejuni* and *C*. *coli* species specific genetic indicators.

						CAMP0952	CAMP0908	CAMP1271
SRR#	SPECIES	ST	CC	SOURCE	YEAR	*mapA*	*hipO*	*ceuE*
**SRR6944251**	*C*. *coli*	7818	828	PM_CHICKEN	2018	3	-	3
**SRR7446975**	*C*. *coli*	7818	828	PM_CHICKEN	2018	3	-	3
**SRR7525348**	*C*. *coli*	7818	828	PM_CHICKEN	2018	3	-	3
**SRR7665237**	*C*. *coli*	7818	828	PM_CHICKEN	2018	3	-	3
**SRR7822697**	*C*. *coli*	7818	828	PM_CHICKEN	2018	3	-	3
**SRR8075046**	*C*. *coli*	7818	828	PM_CHICKEN	2018	3	-	3
**SRR8381509**	*C*. *coli*	7818	828	PM_CHICKEN	2018	3	-	3
**SRR8592680**	*C*. *coli*	7818	828	PM_CHICKEN	2018	3	-	3
**SRR8389990**	*C*. *coli*	7818	828	PM_CHICKEN	2018	3	-	3
**SRR6767587**	*C*. *coli*	7818	828	LA_CHICKEN	2018	3	-	3
**SRR7615056**	*C*. *coli*	7818	828	LA_CHICKEN	2018	3	-	3
**SRR7903399**	*C*. *coli*	7818	828	LA_CHICKEN	2018	3	-	3
**SRR8136396**	*C*. *coli*	7818	828	LA_TURKEY	2018	3	-	3
**SRR10101895**	***C*. *jejuni***	7818	828	RM_CHICKEN	2019	3	-	3
**SRR10101909**	***C*. *jejuni***	7818	828	RM_CHICKEN	2019	161	-	3
**SRR10194298**	*C*. *coli*	7818	828	RM_CHICKEN	2019	161	-	3
**SRR10224765**	*C*. *coli*	7818	828	RM_CHICKEN	2019	161	-	3
**SRR10586091**	*C*. *coli*	7818	828	RM_CHICKEN	2019	3	-	3
**SRR10609015**	*C*. *coli*	7818	828	RM_CHICKEN	2019	161	-	3
**SRR10612208**	*C*. *coli*	7818	828	PM_CHICKEN	2019	161	-	3
**SRR10069420**	*C*. *coli*	7818	828	PM_CHICKEN	2019	3	-	3
**SRR10075995**	*C*. *coli*	7818	828	PM_CHICKEN	2019	3	-	3
**SRR10096962**	*C*. *coli*	7818	828	PM_CHICKEN	2019	3	-	3
**SRR10119163**	*C*. *coli*	7818	828	PM_CHICKEN	2019	3	-	3
**SRR10356106**	*C*. *coli*	7818	828	PM_CHICKEN	2019	3	-	3
**SRR10530298**	*C*. *coli*	7818	828	PM_CHICKEN	2019	161	-	3
**SRR10691777**	*C*. *coli*	7818	828	PM_CHICKEN	2019	3	-	3
**SRR10832065**	*C*. *coli*	7818	828	PM_CHICKEN	2019	3	-	3
**SRR10832070**	*C*. *coli*	7818	828	PM_CHICKEN	2019	3	-	3
**SRR9202000**	*C*. *coli*	7818	828	PM_CHICKEN	2019	3	-	969
**SRR9326349**	*C*. *coli*	7818	828	PM_CHICKEN	2019	161	-	3
**SRR10574881**	*C*. *coli*	7818	828	PM_CHICKEN	2019	-	-	3
**SRR9874561**	*C*. *coli*	7818	828	PM_CHICKEN	2019	-	-	3
**SRR10218554**	*C*. *coli*	7818	828	LA_CHICKEN	2019	3	-	3
**SRR9417598**	*C*. *coli*	7818	828	LA_CHICKEN	2019	3	-	3

(-) indicates no genomic match within the database. *Campylobacter* species specific gene alleles detected via the Oxford PubMLST database for *hipO*, *map*A and *ceuE*.

## Discussion

U.S. policy changes were enforced over the past few years to reduce antibiotic use in the agricultural industry to help decelerate evolving acquired antimicrobial resistance. Still, in 2018, over 6650 tons of medically important antibiotics were sold in the U.S. for use in animals [26]. In 2018, the antibiotics sold in the U.S. with the approval for use in food animals by tonnage was primarily tetracyclines (66%, 4389 tons), penicillin (12%, 789 tons), macrolides (8%, 532 tons), aminoglycosides (5%, 332.5 tons), sulfonamides (5%, 332.5 tons), lincosamides (2%, 133 tons), cephalosporins (1%, 66.5 tons), and fluoroquinolones (<1%, < 66.5 tons) [26]. As human populations continue to rise and increase demands on food animal production, antimicrobial applications to maintain herd health and humane conditions can be expected to further drive microbial evolution and positive selection for AMR pathogens in food animal environments. Our 2018–2019 dataset, which included *Campylobacter* isolates from live food animals, processed poultry and retail meat in North Carolina, detected the highest prevalence of *C*. *coli* and *C*. *jejuni* AMR genes toward tetracycline, beta-lactam, aminoglycoside, macrolide, quinolone and lincosamide antibiotics (in descending order). This closely matches the top U.S. antimicrobial distribution reported for use in food animals with the exception, no sulfonamide resistance genes were detected in this *Campylobacter* dataset. The phenotypic AMR expression of 129 *Campylobacter* isolates (111 live animal and 18 retail meat) showed a slightly different distribution having the highest resistance toward tetracyclines, quinolones, aminoglycosides, lincosamides and macrolides in descending order (beta-lactams were not included in the *Campylobacter* AST panel).

Fluoroquinolones and macrolide antibiotics are the most common medications used in human medicine to combat moderate to severe campylobacteriosis [21–23, 27]. Despite the removal of fluoroquinolone application fifteen years ago, *Campylobacter* quinolone resistance has persisted in poultry populations [28]. In fact, 90.3% (121/134) *gyrA* mutations were from poultry isolates, while only 13 were isolated from swine and cattle. Quinolone resistance in human *Campylobacter* infections has also remained and gradually increased in the past decade, with 39.4% *C*. *coli* and 27.75% *C*. *jejuni* infections resistant to ciprofloxacin and nalidixic acid in 2017 [29].

The overall quinolone resistance in this dataset, 25.7% (88/343) *C*. *coli* and 23.3% (46/198) *C*. *jejuni*, align closely to previous year’s phenotypic quinolone resistance trends reported by the NARMS Now Integrated Data, with the exception of turkey *C*. *coli* isolates [27, 29]. The NC 2018–2019 *C*. *coli* from turkey had a higher *gyrA* mutation prevalence of 64% in live animals and 75% in processed meat compared to the 2017 NARMS average *C*. *coli* phenotypic quinolone resistance in live turkeys and processed turkey meat, which had 33% and 37% resistance respectively [29]. The *gyrA* mutation causes variation in DNA gyrase and reduces the affinity of fluoroquinolone antibiotics to bind to the enzyme. All *gyrA* mutations were located at codon 86 with 97% (130/134) *ACA-> ATA* coding for Thr-86-Ile (T86I) and four *C*. *jejuni* mutations *ACA->GCA* coded for Thr-86-Ala (T86A). The Thr-86-Ile shift is the most commonly observed mutation in fluoroquinolone resistant *Campylobacter* isolates and confers high-level resistance to ciprofloxacin [28, 30].

The prevalence of macrolide and lincosamide resistance among *C*. *coli* in NC 2018–2019 isolates, predicted by the presence of point mutation *23S (A2075G*), was higher than NARMS phenotypic resistance prevalence in previous years [27, 29]. In our study, all isolates containing 23S (A2075G) expressed high resistance to azithromycin, erythromycin, and clindamycin in the MIC susceptibility testing. This single nucleotide substitution in the 23s rRNA sequence disrupts the ribosomal structure which interferes with macrolide and lincosamide binding sites on the 50s rRNA, which is the main mechanism of action for both drug classes to prevent bacterial protein synthesis [31, 32]. NC 2018–2019 macrolide and lincosamide resistance in *C*. *coli* from turkey was two times higher than reported for the U.S. in 2017.

Since NARMS testing began in 1997, the prevalence of *C*. *jejuni* macrolide resistance has remained low (below 4%) [27, 33]. Our results disclosed this same low-level macrolide resistance trend in *C*. *jejuni*. One possible causation was the lack of the 23S rRNA point mutation among *C*. *jejuni* isolates, which alters the antibiotic binding site, conferring macrolide resistance. The 23S (A2075G) mutation is known to reduce the ability of *C*. *jejuni* to colonize hosts [27]. The mutation does not significantly impact *C*. *coli* fitness resulting in the observations detailed above.

50S L22 mutations were also detected in this data set, primarily in *C*. *jejuni*, but as the MIC sensitivity test results show in Figs 1 and 2, the 50S L22 (A103V) substitutions alone did not result in phenotypic resistance to azithromycin or erythromycin. Previous studies found this ribosomal L22 (A103V) point mutation in *Campylobacter* isolates with high resistance to erythromycin, although it was suspected the mutation alone had minimal direct involvement in the resistance phenotype [34, 35]. This L22 mutation was shown to act synergistically with the CmeABC efflux pump to cause macrolide resistance [24, 27, 34, 36]. All isolates in this study carrying the *50S L22* mutation also contained *cmeABC* and *cmeR* (with variable percent genomic identity), indicating these carriers may have the potential to mount macrolide resistance under certain conditions.

The *cmeABC* sequence codes for the multidrug efflux complex located in the bacterial membrane. The presence of this efflux pump is essential for *Campylobacter* colonization in animal intestines which explains its presence in the isolates in this dataset [34]. The efflux pump can prompt resistance to structurally diverse compounds such as macrolides, quinolones, tetracyclines, ethidium bromide, and various detergents [37]. The *cmeABC* and *cmeR* outer membrane multidrug efflux pump components in this study were identified with ≥90% coverage and ≥70% identity. *CmeR*, codes for the CmeR repressor which binds to the cmeABC promoter region to regulate activity [38], had greater percent identity variability and trends were noted by species, *C*. *coli* and *C*. *jejuni*. *Campylobacter spp*. are known to have high sequence variation for outer membrane proteins. Mutations in the CmeR-binding site and the presence of the RE-CmeABC variant are both linked to enhanced antibiotic resistance in *Campylobacter* [27, 30, 37]. The RE-CmeABC variant was found in a previous study to be increasing among food animals and more common within *C*. *jejuni* compared to *C*. *coli* [39]. Further research is needed to identify variants and determine the implications of the efflux pump molecular variability observed in this study.

The most important mechanism of aminoglycoside resistance in *Campylobacter spp*. is enzymatic modification [40]. In this dataset, *C*. *jejuni* isolates primarily carried 2 aminoglycoside resistance genes, *aph7* and *aph(3’)-III* while *C*. *coli* contained a variety of aminoglycoside resistance genes, coding for 8 different enzymes and 2 point mutations, that alter the drug ribosomal binding site. The *C*. *coli rpsL* point mutations were all a sequence change *AAA->AGA* resulting in the translation substitution Lys->Arg, with the majority occurring at codon 43 (*rpsL(K43R)*) and two occurring at codon 88 (*rpsL(K88R))*. Both amino acid substitutions cause structural changes to the aminoglycoside binding site [41]. The MIC susceptibility tests showed high gentamicin resistance in isolates containing AMR gene *aph(2’)-IF*. The highest prevalence of *aph(2’)-IF* in this dataset was *C*. *coli* carried in 57% live turkey and 70% processed turkey. The prevalence *aph(2’)-IF* conferring resistance to gentamicin in *C*. *coli* turkey isolates is higher than the 2017 NARMS findings of 11% and 58% *C*. *coli* phenotypic gentamicin resistance in live and processed turkey respectively [29].

Tetracycline and beta-lactam drug class resistance had the highest overall prevalence in this *Campylobacter* dataset. The high prevalence of beta-lactam antimicrobial resistance genes demonstrates *Campylobacter* as a resistome reservoir for diverse AMR genotypes to penicillin-type drugs (*blaOXA*, *blaOXA-61*, *blaOXA-157*, *blaOXA-184*, *blaOXA-193*, *blaOXA-460*, *blaOXA-465*, *blaOXA-489*, *blaOXA-577*, *blaOXA-578*, *blaOXA-594*, and *blaOXA-603)*.

DNA sequence-based analysis is reported to have better precision at identifying bacterial AMR compared to phenotypic testing; however, the MIC susceptibility testing resulted in slightly greater phenotypic resistance than genotypically predicted for a few drug classes [42]. Mainly, *Campylobacter* isolates from swine (4 *C*. *coli* and 1 *C*. *jejuni*) plus one novel ST *C*. *jejuni* isolated from chicken expressed resistance to clindamycin without detectable lincosamide AMR genes. Also, two additional swine *C*. *coli* isolates expressed resistance to ciprofloxacin and nalidixic acid while quinolone AMR genes were not detected. These isolates with a phenotype-genotype AMR mismatch were of varying ST, non-clonal. This finding could be due to novel recombination of AMR genes, or the influence of the cmeABC efflux not fully characterized in this study.

MDR pathogens are a public health concern in the food chain as they often pose increased mortality risk and can be more expensive to treat due to prolonged extensive therapy [43]. The 43.1% predicted MDR among NC 2018–2019 *Campylobacter* isolates (molecular MDR 41.4% *C*. *coli* and 22% *C*. *jejuni*) was also higher than the 2017 NARMS phenotypic MDR prevalence for food animal, processing and retail meat MDR (*C*. *coli* isolates <33% and *C*. *jejuni* <3%) [29, 43]. In this dataset, there was no significant difference in total *Campylobacter* AMR and MDR prevalence from 2018 to 2019. Many unique MDR profiles were observed somewhat sporadically between 2018 and 2019. Molecular MDR was three times more likely to occur in *C*. *coli* isolates compared to *C*. *jejuni*. Too few isolates contained the same combination of MDR genes to prove significant trends. There was an increase in MDR profile diversity in 2019; however, the increase was not significant (p = 0.78). The variable results demonstrate the complexity in genomic MDR combinations as AMR genes can be on mobile genetic elements such as plasmid DNA, transposons, or integrons and transfer genes from one another by conjugation, transduction, and transformation [43].

Turkeys have been reported to harbor the highest prevalence of MDR *Campylobacter* compared to the other food animals in this study which parallels findings of many other studies in the U.S. and around the world [43–45]. While *C*. *coli* were abundant in turkey live animal and processing isolates, only one *C*. *coli* was isolated from retail turkey meat which raises suspicion to the fitness of *C*. *coli* in meat storage or packaging conditions. A similar observation was noted in chicken processing, where *C*. *coli* dominated live animal and processing facility isolates but became the minority species isolated from retail products. *C*. *jejuni* may have greater fitness or survivability through poultry processing conditions. Further research is warranted to evaluate this presumption as it pertains to North Carolina poultry production facilities.

*C*. *jejuni* adapted to evade the human immune system with virulence factors *neuABC* and *cst-II* or *cst-III*, which code for LOS cell-surface structures that mimic human gangliosides [46, 47]. Infection with *C*. *jejuni* strains that express ganglioside-like LOS can cause development of antibodies to the ganglioside like LOS that cross-react with natural gangliosides abundant in the human nervous system. This autoimmune escalade leads to nerve demyelination, loss of signal transduction, resulting in the loss of motor function that characterizes GBS [46, 48]. *C*. *jejuni* is the most common etiological agent for GBS [27, 49, 50]. In our study, the LOS virulence factors were detected primarily in *C*. *jejuni* isolates from chicken (at all three stages of production) and cattle. We found 57.6% *C*. *jejuni* in NC meat processing have the potential to trigger GBS. A previous *in silico* study conducted in the U.S. evaluated 827 *C*. *jejuni* from the FDA GenomeTrakr SRA Database (Project PRJNA258021), isolates collected between 2002–2017 from food, environmental samples and human clinical cases, had found 43.7% isolates contained *cst-II* while only 9.4% contained *cst-III* [48]. Our study identified a higher prevalence of *cst-III* at 26%, which correlated with specific ST in chicken and cattle, and a *cst-II* prevalence of 30.6% that had a more generalized distribution, spanning many ST and both *Campylobacter spp*. *Cst-II* was identified in *C*. *jejuni* from turkey and swine and within three *C*. *coli* isolates. *C*. *jejuni*-like LOS locus within *C*. *coli* is reportedly rare and is an indication of introgression between the two species [51].

MLST minimum spanning tree showed the ancestral linkage and evolution within this dataset based on the allele sequences from seven housekeeping genes, which are highly conserved essential genomic segments. The ST with at least four matching alleles are grouped into a CC [10]. In this dataset, the 78 *C*. *coli* ST were linked to two CC (CC 828 and CC 1150) which are the only two MLST defined CC associated with *C*. *coli* in agriculture and clinical isolates [10]. The 66 *C*. *jejuni* ST in this dataset were linked to 21 CC. Characteristic high rates of horizontal gene transfer (HGT) among *C*. *jejuni* often leads to a more nonclonal population structure organized by CC of related isolates [10]. *C*. *jejuni* host specialism was observed in this dataset, with 94% cattle ST falling within CC 21, CC61, and CC 42, which matches the findings of a recent study where cattle *C*. *jejuni* ST specialists were identified to be within CC 21 and CC61 [52]. The retail meat *Campylobacter spp*. isolates able to remain culturable through adverse processing conditions included 30 different ST (100% *C*. *coli* ST belonged to CC 828 and *C*. *jejuni* ST belonged to 11 different CC). Retail meat ST were clonal to 77% ST isolated in earlier stages of poultry production. These ST show adaptability and fitness in adverse environments. The majority of genotypes isolated from human Campylobacteriosis cases have also been isolated from food animals [10, 53].

The coexistence of *C*. *coli* and *C*. *jejuni* under the same environmental pressures within the food animal production system provides a great opportunity for interspecies genomic exchange. Several findings in this study provided evidence of such progressive genomic exchange, which may impede some diagnostics that rely on historically declared species-specific gene targets. Through MLST analysis, two *C*. *jejuni* collected in 2019 from chicken retail meat were found to be clonal with *C*. *coli ST 7818*, *CC 828*. The *C*. *jejuni* and *C*. *coli* ST 7818 isolates also shared the same alleles for *mapA* and *ceuE* genes, which are often used to decipher the two species, while the *C*. *jejuni* ST 7818 isolates lacked the *hipO* gene that is often targeted for *C*. *jejuni* detection in routine diagnostics such as real-time PCR [11]. Three *C*. *coli* isolates from live and processed chicken also contained the *C*. *jejuni*-like LOS locus *neuABC* and *cstIII*, which is an adaptation to evade the human immune system [46, 47]. The presence of this *C*. *jejuni*-like LOS locus in *C*. *coli* was reportedly rare and is an indication of introgression between the two species [51, 54, 55]. Genomic transfer of large segments of DNA can introduce a high number of polymorphisms and create novel phenotypes rapidly [10]. This high rate of HGT was evident in this study by the significant increase in untypeable sequences from 2018 to 2019 due to the genomic variation in the seven highly conserved housekeeping loci. The MLST database has greater coverage of the 2018 isolates; therefore, we cannot determine in this study whether the rate of genomic variation is continuous or fluctuating over time. This should be further evaluated in the food production systems as *Campylobacter* species continue to evolve together under the same environmental pressures. Of concern, *C*. *coli* generally carries greater multidrug resistance while *C*. *jejuni* has adapted as a more effective human pathogen, causing 90% of human Campylobacteriosis cases. High levels of interspecies genomic exchange between these two species may alter pathogen survivability, resistance trends, and could also pose a greater risk to public health.

In this study, we identified evidence of an increase in antimicrobial resistance, virulence factor distribution, and interspecies genomic exchange between *C*. *coli* and *C*. *jejuni* in NC food animal production. As *Campylobacter* continues to be the worldwide leading cause for foodborne illness, further research is needed to track evolutionary patterns and interspecies genomic exchange, which may alter sensitivity and specificity of diagnostic tests and aid *Campylobacter* survivability and transmission through food processing. WGS and open access bioinformatic tools prove to be effective in identifying trends and allow for detailed analysis, surveillance, and deeper interpretations despite high rates of HGT.

## Methods

### Data collection

The *C*. *coli and C*. *jejuni* data collection was conducted in accordance with the NARMS surveillance and laboratory protocols in North Carolina [56]. The timeframe of this study was from January 2018 through December 2019. During this time, retail meat samples, totaling 30 packages of fresh (never frozen) poultry products, were collected twice a month from a variety of grocery stores throughout North Carolina. The FDA pre-assigned grocery store sampling locations using the chain store guide https://www.chainstoreguide.com/ to identify all grocery stores within a 50 mile radius from the NCSU Molecular Epidemiology Laboratory based on zip codes. The FDA divided the sampling sites geographically into quadrants and used a random number generator to randomly assign the order to be sampled. The FDA randomized list of grocery stores is updated for each year [57]. Caution was taken during sample collection to select a variety of brands and products from multiple sources. The biweekly sample set included 20 packages of chicken (bone-in/skin-on) and 10 packages of ground turkey.

The retail meat isolates collected from 2018 to 2019 were included in a cross-sectional study to determine the prevalence of AMR, MDR, virulence genes, and phylogenomic relationship among *C*. *coli and C*. *jejuni* within food animals, poultry production facilities, and retail meat in North Carolina. The USDA conducts live food animal surveillance testing by sampling cecal contents of healthy swine, cattle, chickens and turkeys in FSIS-regulated livestock and poultry slaughter establishments. Poultry production isolates obtained by the USDA were procured by testing chicken and turkey carcasses through PR/HACCP verification samples from poultry slaughter establishments in North Carolina [57]. NARMS sampling plan lists establishments in a tiered, randomized fashion based on slaughter volume, in order to have a representative sampling scheme covering production at the national level [57].

For this study, NARMS *C*. *coli* and *C*. *jejuni* isolates collected in North Carolina by the USDA, FDA, and NCSU CVM Thakur Molecular Epidemiology Laboratory from 2018 to 2019 were identified in the National Center for Biotechnology Information (NCBI) database https://www.ncbi.nlm.nih.gov under bioprojects PRJNA292664, PRJNA292668, and PRJNA287430. Forward and reverse sequencing reads for 541 *C*. *coli* and *C*. *jejuni* isolates from live chicken, turkey, swine and cattle (n = 198), poultry carcasses at production facilities (n = 291), and retail meat (n = 53) were obtained for further AMR, virulence factor, and phylogenic analysis (Table 3).

**Table 3:** North Carolina 2018–2019 *Campylobacter spp*. study dataset distribution by source.

	LA- Chicken	PM- Chicken	RM- Chicken	LA- Turkey	PM- Turkey	RM- Turkey	LA- Cattle	LA- Swine	Total
*C*. *coli*	38	162	16	35	20	1	5	66	**343**
*C*. *jejuni*	33	108	36	0	1	0	16	4	**198**
**Total**	**71**	**270**	**52**	**35**	**21**	**1**	**21**	**70**	**541**

*C*. *coli* and *C*. *jejuni* isolates collected in 2018 and 2019 throughout North Carolina. Chart coding: Live Animal (LA), Processed Meat (PM), Retail Meat (RM).

### Isolation and identification of *Campylobacter* isolates

Samples were stored in refrigeration at 4°C and processed within 96 hours after purchase. For sample processing, 50g of retail meat was aseptically placed in 250 ml buffered peptone water (BPW) and then placed in a mechanical shaker at 200 rpm for 15 minutes. After, 50 ml BPW rinse was added to 50 ml double strength (2x) Bolton broth and incubated in a microaerophilic atmosphere (85% N2, 10% CO2, 5% O2) for 24 hours. The inoculum was plated on Campy Cefex Agar and incubated under the same conditions for 24 hours. One *Campylobacter* colony was selected from each positive sample, plated on blood agar and incubated for another 24 hours. *Campylobacter* confirmatory testing was conducted by gram stain, oxidase and catalase tests before further processing. *Campylobacter* samples were placed in Brucella broth with 15% glycerol mixture and stored at -60-80°C. Frozen samples were shipped on dry ice to the FDA for species confirmation, AST, and WGS [58]. WGS for isolates collected July-Dec 2019 was conducted in the NCSU Thakur Molecular Epidemiology Laboratory with the Illumina MiSeq platform following the FDA NARMS protocol [56].

### DNA isolation and WGS

DNA isolation was conducted using a modified version of the Qiagen DNeasy Blood and Tissue kit (Qiagen, Venlo, Netherlands). Approximately 1μl of cells (one pure *Campylobacter* isolate per sample) were added to 360 μl of Buffer ATL in a 1.5 ml microcentrifuge tube and allowed to sit at room temperature to incubate. During cell lysis, the samples were vortexed with a Fisher variable vortex at medium speed (7) for 5–10 seconds before adding 40 μl Proteinase K. Tubes were inverted 3–5 times and vortexed again for 5–10 seconds. Samples were incubated at 56°C for 1 hour while shaking at 600 rpm. After incubation, samples were again vortexed briefly before adding 8 μl of RNase A. Tubes were inverted 3–5 times, vortexed for 5–10 seconds and left to incubate at room temperature for 3–5 minutes. AL buffer was preheated in a 30°C water bath, then 400 μl was added to each sample and inverted 3–5 times. Samples were vortexed for 10–12 seconds before adding 400 μl of 95–100% ethanol. Tubes were again inverted and vortexed. DNA was collected in a DNeasy spin column by centrifugation at 10,000 rpm during the final wash steps. A pre-warmed 10 mM Tris-HCl (pH 8.0) (Fisher Scientific, Waltham, MA) solution was used as the elution buffer for each DNA sample. A quality check for each sample was conducted using the Nanodrop 2000 Spectrophotometer. The high-sensitivity assay kit for the Qubit 4.0 Fluorometer was used to verify the concentration of double-stranded DNA (ThermoFisher Scientific, Waltham, MA).

After DNA quantification, DNA libraries of each sample were prepared for WGS using a Nextera XT kit (Illumina, San Diego, CA). Nextera XT DNA Sample Prep Kit (Illumina Inc., San Diego, CA) was used to process 0.3 ng/μl DNA from each isolate. DNA was pooled together and sequenced on an Illumina MiSeq (Illumina, San Diego, CA) using 2 x 250 or 2 x 300 paired-end reads. WGS reads were demultiplexed and submitted to the NCBI database where they were given an accession number.

### SRA assembly and BLAST

Genome sequences were assembled *de novo* with Shovill version 1.1.0 using SPAdes version 3.13.1 [59, 60]. The assembled genomes were then passed through a bioinformatic pipeline using BLAST techniques to identify AMR genes, virulence factors, and AMR associated point-mutations from multiple databases within the programs ABRICATE v. 0.9.9 and STARAMR. The ABRICATE databases utilized in this study included the CARD, ResFinder, ARG-ANNOT, NCBI AMRfinderPlus, and VFDB [38, 61–66]. STARAMR databases included Pointfinder v050218, Resfinder v050218.1, MLST v2.18.0, Plasmidfinder database date 25 Oct 2019 [67, 68]. Genome assembly quality was assessed using QUAST version 5.0.2 [69]. Point mutations and AMR genes were cross referenced with the updated NCBI Pathogen Detection database. Molecular MDR was defined as the presence of AMR genes conferring resistance to three or more antibiotic drug classes out of the combined ABRICATE databases, which contain 10 main drug classes (aminoglycosides, beta-lactams, lincosamides, ketolides, macrolides, phenicols, quinolones, sulfonamides, tetracyclines, and trimethoprim-sulfamethoxazole) relevant to this study.

The VFDB did not contain a *cst-II* reference genome and therefore failed to detect this gene in the dataset. As an alternate approach, BLAST function in the CLC Genomics Workbench v11.0 was used to screen *Campylobacter* isolates positive for *neuABC* genes for *cst-II* >90% identify using *C*. *jejuni* RM3196 (GenBank CP012690.1) as a reference sequence for *cst-II* [48].

Assembled genomes were submitted to the PubMLST database to verify ST and to determine CC [53]. ST were based on the sequence diversity within seven conserved loci, referred to as housekeeping genes or the allelic profile. The *Campylobacter spp*. allelic profile consists of loci: aspA, glnA, gltA, pgm, tkt, uncA. Isolates within the same ST have identical sequences at all seven loci and are deemed members of a single clone. Bacteria in a CC contain the same alleles for at least four of the seven loci with at least one other ST in the group [57]. Untypeable ST were submitted to the Bacterial Isolate Genome Sequence Database (BIGSdb) for curation and verification [70]. MLST data was used to generate the Global Optimal eBurst matrix using PHYLOViZ software [71]. The all loci search function in the pubMLST database was used to identify *mapA*, *ceuE* and *hipO* alleles for assembled genomes [53].

### Antimicrobial susceptibility testing (AST)

USDA and FDA NARMS laboratories conducted broth microdilution assays using the Sensititre^™^ CAMPY panel to determine the MIC for each isolate against 10 antimicrobial agents (Gentamicin, Telithromycin, Clindamycin, Azithromycin, Erythromycin, Chloramphenicol, Florfenicol, Ciprofloxacin, Nalidixic acid, and Tetracycline) using the NARMS interagency laboratory protocol [58]. Antimicrobial susceptibility cutoff values were determined based on current NARMS program standards [72]. Phenotypic MDR was defined as resistance to three or more antibiotic drug classes out of seven drug classes tested on the Sensititre^™^ CAMPY plate (aminoglycosides, ketolides, lincosamides, macrolides, phenicols, quinolones, and tetracyclines).

### Statistical analysis

Tests for statistical analysis included the use of contingency tables to estimate odds ratios and confidence intervals for the odds ratio. The odds ratio is a measure of likelihood indicating association, its direction, and magnitude (odds ratio and confidence interval should not include 1). Prevalence was determined by dividing the number of isolates containing the gene by the total number of isolates. Statistically different values possessed P-values of ≤ 0.05. These analyses were computed utilizing SAS analytics and MedCalc software, version 19.2.1 [73, 74].

## Supporting information

S1 Fig Prevalence of antimicrobial resistance (AMR) and multidrug resistance (MDR) for *C*. *coli* and *C*. *jejuni* by source. Prevalence was computed by dividing the number of AMR or MDR isolates by the total number of *C*. *coli* or *C*. *jejuni* isolated from each source. Chart source coding: Live Animal (LA), Processed Meat (PM), Retail Meat (RM).
(TIF)

S1 Table *C*. *coli* and *C*. *jejuni* multidrug resistant genotypic profiles. Identified genetic sequences are >95% identity and coverage.
(TIF)

S2 Table *C*. *jejuni* isolates with virulence factors *neuABC* and *cst-II* associated with LOS glycan mimicry. Virulence factors *neuABC* were determined by BLAST against the VFDB. *Cst-II* was detected by BLAST using the RM3196 reference sequence within the CLC Genomic Workbench ≥ 90% coverage and identity. Virulence factor positive isolates are listed by source, sequence type (ST), and clonal complex (CC). ST prevalence equals the total number of ST isolates containing *neuABC* and *cst-II* divided by the total of the specific ST identified in this dataset. Chart source code: Retail Meat (RM), Live Animal (LA), Processed Meat (PM). (*) *neuA1* coverage range between 25–40%.
(TIF)

S3 Table *C*. *jejuni* isolates with virulence factors *neuABC* and *cst-III* associated with LOS glycan mimicry. Virulence factors *neuABC* and *cst-III* were determined by BLAST against the VFDB. Virulence factor positive isolates are listed by source, sequence type (ST), and clonal complex (CC). ST prevalence equals the total number of ST isolates containing *neuABC* and *cst-III* divided by the total of the specific ST identified in this dataset. Chart source code: Retail Meat (RM), Live Animal (LA), Processed Meat (PM).
(TIF)

S4 Table Dataset Sequence Read Archive (SRA) list. Sequence reads from NCBI Bioprojects PRJNA292664, PRJNA292668, and PRJNA287430 used in this study.
(TIF)
