# Supplementary figures and images for: Antimicrobial resistance and interspecies gene transfer in Campylobacter coli and Campylobacter jejuni isolated from food animals, poultry processing, and retail meat in North Carolina, 2018–2019
Source: PLoS One. 2021 Feb 11;16(2):e0246571. doi: 10.1371/journal.pone.0246571 (PMC7877606; doi:10.1371/journal.pone.0246571)

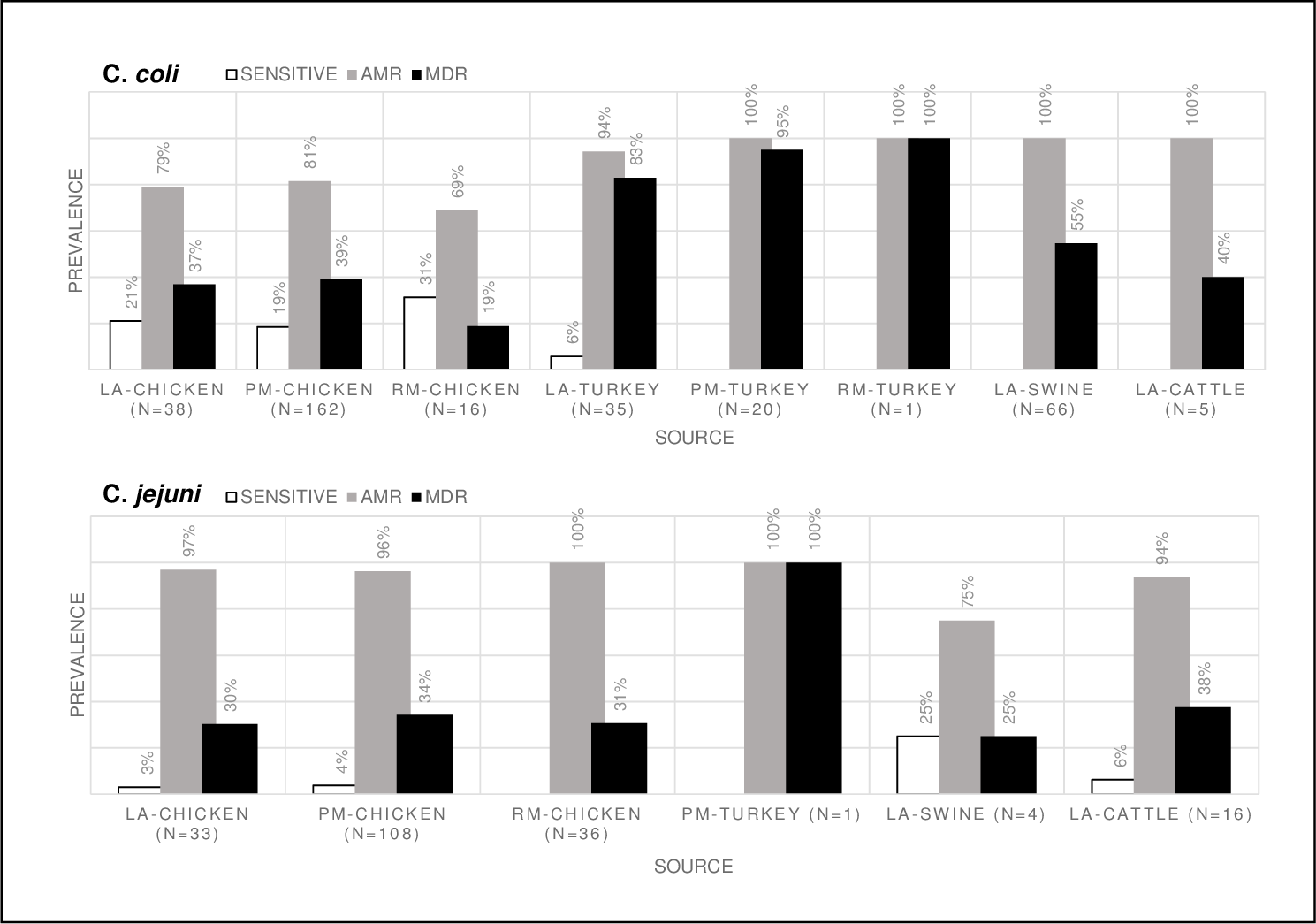

Supplement: S1 Fig — Prevalence was computed by dividing the number of AMR or MDR isolates by the total number of C. coli or C. jejuni isolated from each source. Chart source coding: Live Animal (LA), Processed Meat (PM), Retail Meat (RM). (TIF) [file pone.0246571.s001.tif]

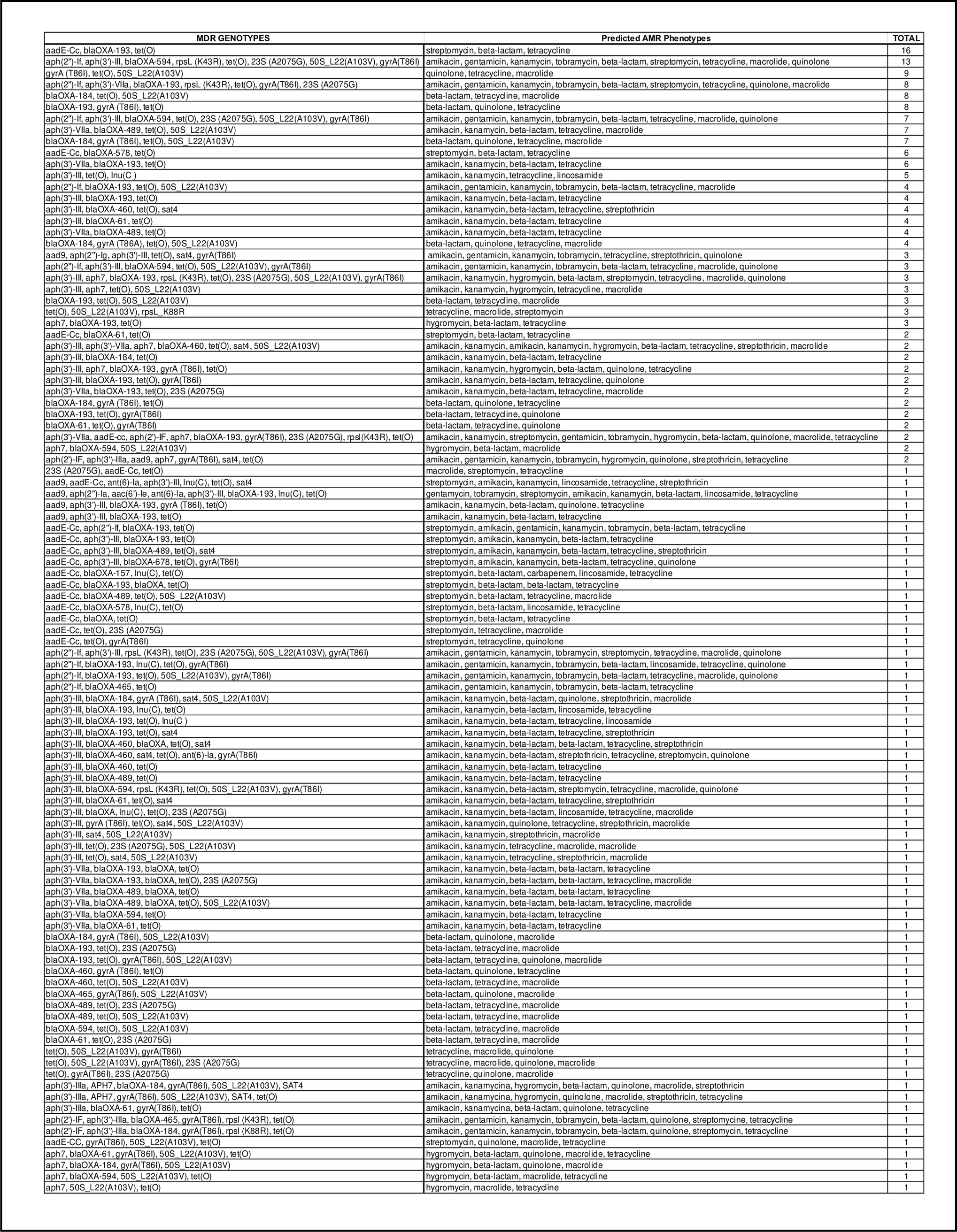

Supplement: S1 Table — Identified genetic sequences are >95% identity and coverage. (TIF) [file pone.0246571.s002.tif]

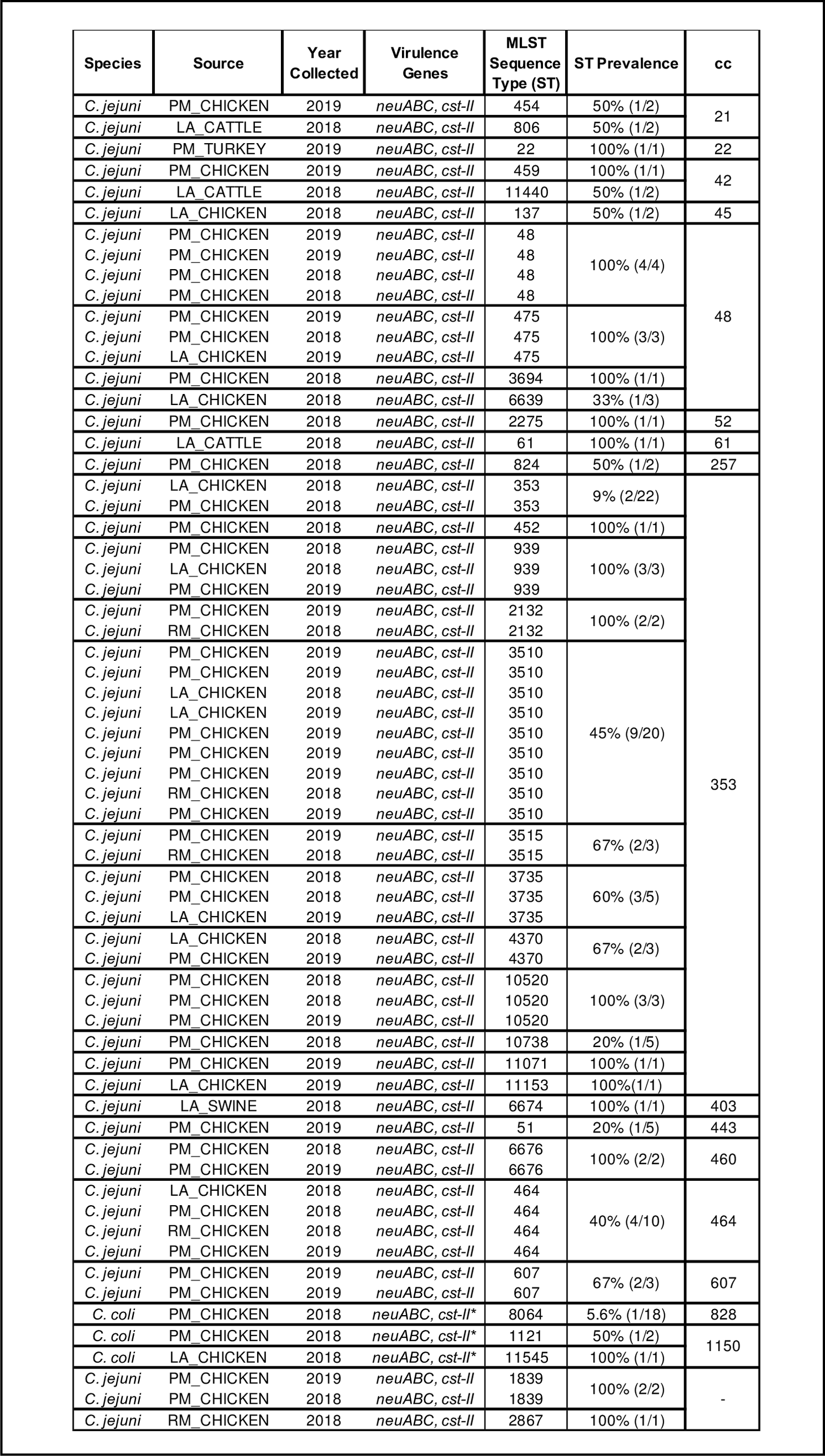

Supplement: S2 Table — Virulence factors neuABC were determined by BLAST against the VFDB. Cst-II was detected by BLAST using the RM3196 reference sequence within the CLC Genomic Workbench ≥ 90% coverage and identity. Virulence factor positive isolates are listed by source, sequence type (ST), and clonal complex (CC). ST prevalence equals the total number of ST isolates containing neuABC and cst-II divided by the total of the specific ST identified in this dataset. Chart source code: Retail Meat (RM), Live Animal (LA), Processed Meat (PM). (*) neuA1 coverage range between 25–40%. (TIF) [file pone.0246571.s003.tif]

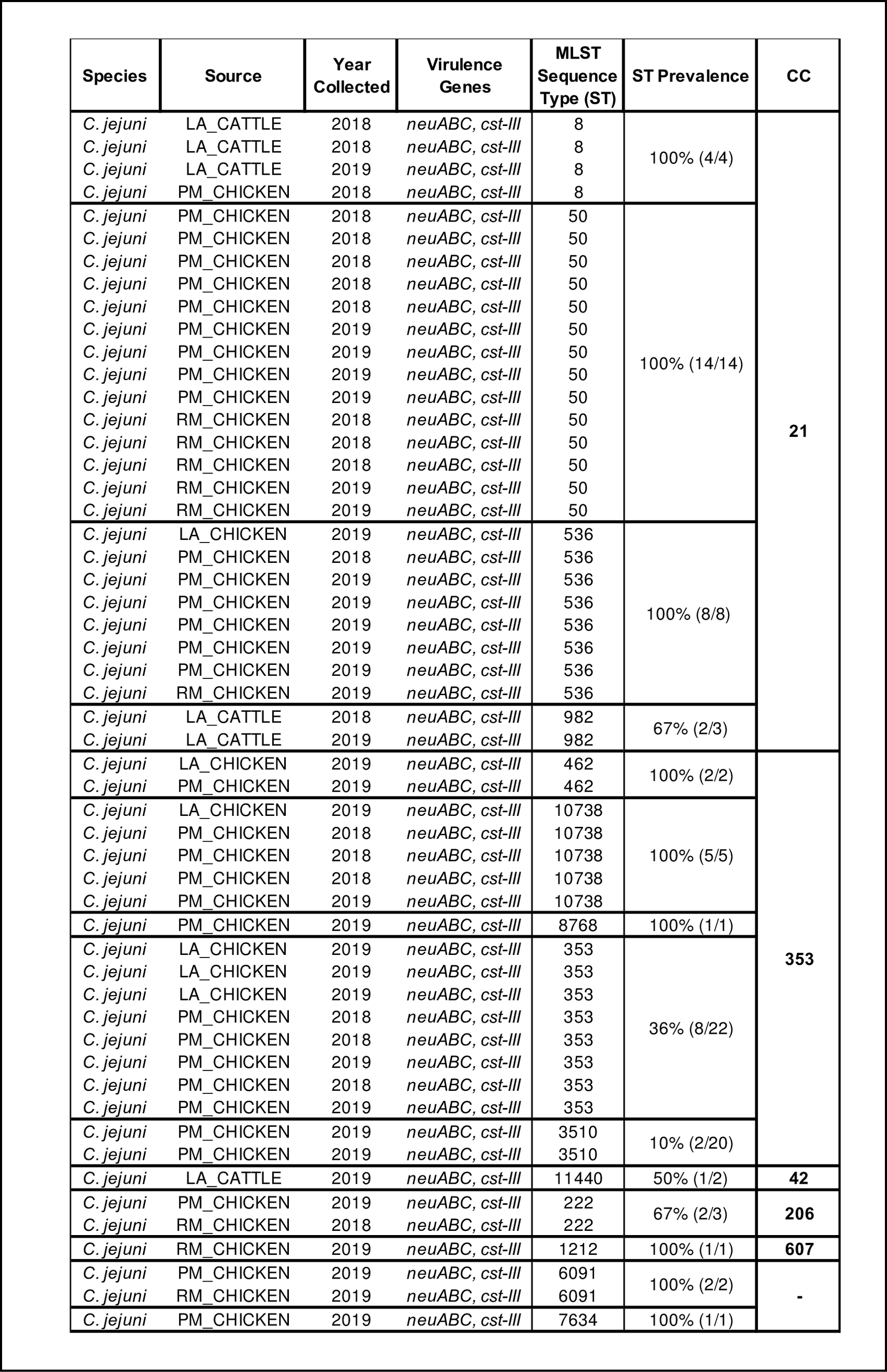

Supplement: S3 Table — Virulence factors neuABC and cst-III were determined by BLAST against the VFDB. Virulence factor positive isolates are listed by source, sequence type (ST), and clonal complex (CC). ST prevalence equals the total number of ST isolates containing neuABC and cst-III divided by the total of the specific ST identified in this dataset. Chart source code: Retail Meat (RM), Live Animal (LA), Processed Meat (PM). (TIF) [file pone.0246571.s004.tif]

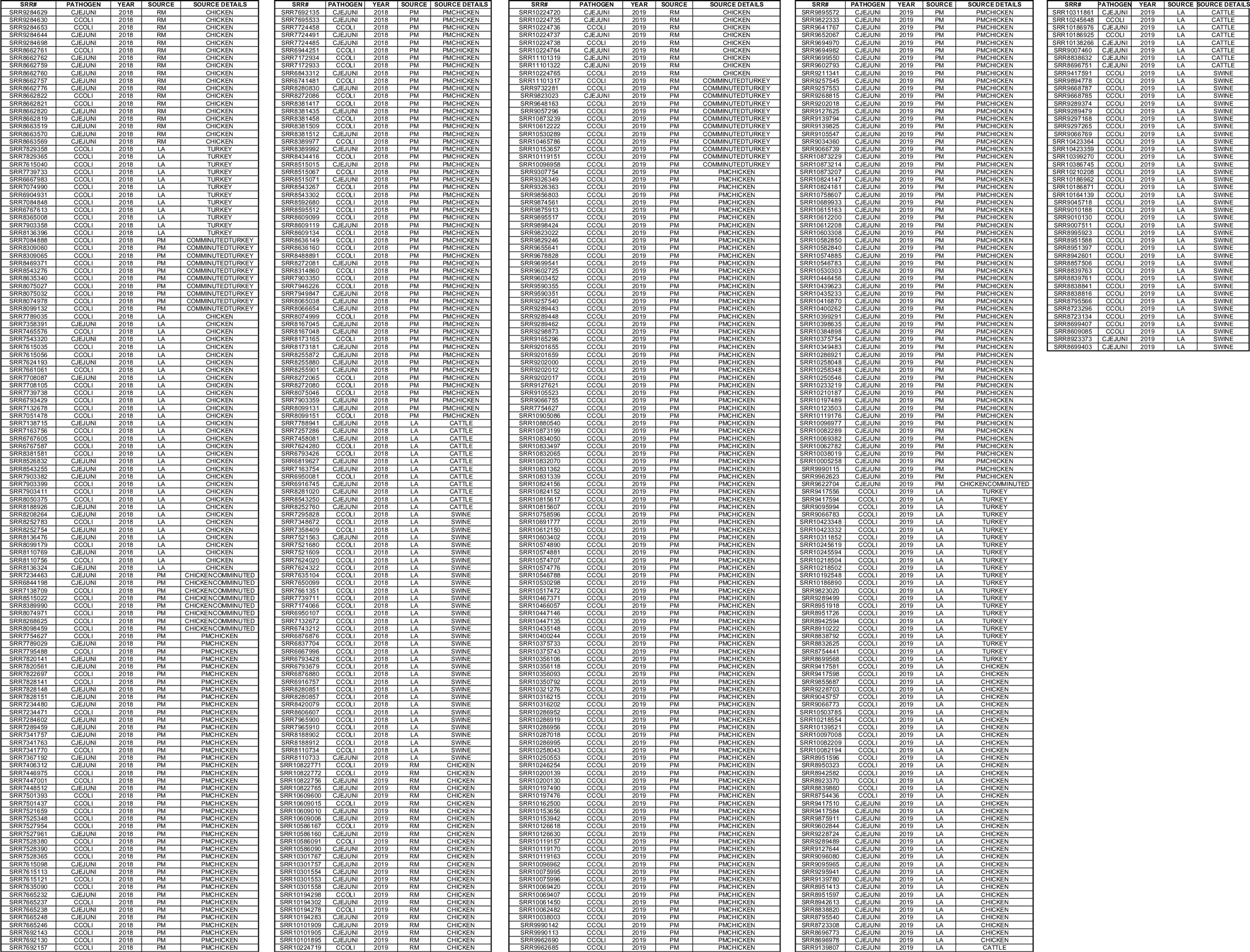

Supplement: S4 Table — Sequence reads from NCBI Bioprojects PRJNA292664, PRJNA292668, and PRJNA287430 used in this study. (TIF) [file pone.0246571.s005.tif]
